# Properties of standing balance control under noisy galvanic vestibular stimulation

**DOI:** 10.3389/fneur.2025.1500308

**Published:** 2025-04-15

**Authors:** Motomichi Sonobe, Tsubasa Mitsutake

**Affiliations:** ^1^Department of Intelligent Mechanical Systems Engineering, Kochi University of Technology, Kochi, Japan; ^2^Clinical Research Center, Saga University Hospital, Saga, Japan

**Keywords:** standing posture, noisy galvanic vestibular stimulation, center of mass, head acceleration, joint strategy, force platform, inertial sensor

## Abstract

Vestibular sensation contributes to balance control during standing as well as somatosensation and vision. Previous studies have indicated that noisy galvanic vestibular stimulation (nGVS) activates vestibular function and improves standing balance in many subjects. However, the mechanism for improving balance control with the addition of nGVS remains unclear. This study aimed to clarify the balance control mechanism improved by nGVS using detailed motion data during quiet standing. Thirty-two young healthy subjects performed quiet standing tests for 40 s with their eyes closed under sham and optimal intensity stimulation. Detailed data consisting of the center of mass (COM) displacement and acceleration of the body, head acceleration, and lower and upper body accelerations were obtained from measurements using a force platform and a head inertial sensor based on the equations of motion of rigid body models. In addition, our study discusses the contributions of joint strategies for COM control and head acceleration control. The contributions of the ankle and hip strategies were calculated from the COM accelerations of the lower and upper bodies. The results indicated that the more effective group of nGVS suppressed head acceleration using the ankle strategy in the anteroposterior direction. This implies that acceleration feedback from vestibular function affects the quality of the ankle joint strategy control. The findings of this study could contribute to the evaluation of vestibular sensory weighting during standing and development of intervention methods for vestibular function using nGVS.

## 1 Introduction

Vestibular sensation, vision, and somatosensory perception are combined to estimate posture for balance control, and the contribution of each organ varies depending on the environment (1). While somatosensory function is dominant during standing, vestibular function also contributes to balance control (2). Vestibular function impairment has been reported to increase the fall rate (3, 4); in particular, patients with bilateral vestibulopathy (BVP) have less balance when their eyes are closed (5). To improve vestibular function, vestibular rehabilitation is expected to increase the weight of the vestibular function and improve balance stability.

Recently, noisy galvanic vestibular stimulation (nGVS) has been investigated as a method for vestibular rehabilitation that noninvasively activates the bilateral vestibular cortex by applying a small electric current from electrodes placed on the mastoid and is expected to be an intervention for BVP (6). It activates the insular peripheral region (7) and contributes to the improvement in standing balance when superimposed on cerebellar transcranial direct current stimulation (8). The stimulation improves standing balance because the threshold for the detection of acceleration by otoliths is reduced (9), and a lower sensory threshold results in improved balance (10). The effectiveness of nGVS in improving the standing balance depends on the environment. Previous studies have reported that body sway is reduced while standing on foam (11, 12) and head acceleration is suppressed when the support surface sways at a sinusoidal wave of 1.2 Hz rather than at lower frequencies (13).

The application of nGVS in patients with BVP, older adults, and young healthy individuals has been reported to improve balance. In patients with BVP, nGVS decreased the center-of-pressure (COP) sway (14–16) and sensory threshold (17). For older adults, decreases in the COP sway on foam (11, 18) and COP trajectory length on hard floors (19) have been reported. In contrast, there is a difference in opinion regarding the effect on young healthy individuals, which is divided between a significant difference on a hard floor (20), no significant difference (21), and a significant difference only in closed-eye standing on foam (12). The significant differences in young healthy individuals could be caused by differences in the frequency and intensity settings of nGVS (22). Among all the aforementioned studies, a common finding was that not all subjects improved their balance with nGVS.

When trying to improve vestibular function by applying nGVS to BVP or the elderly, there are three issues. First, there are differing opinions about which subjects respond well to nGVS, and the general effectiveness of nGVS is also unclear. Second, the process of balance control improvement caused by nGVS is unknown. While the general balance evaluation method has been shown by a decrease in COP trajectory velocity and COP sway area (23), more detailed balance movement information is needed to investigate the impact of vestibular thresholds improved by nGVS on balance strategies (24). Third, it is necessary to identify in advance which subjects will respond well to nGVS. A previous study suggested that subjects who respond well to nGVS have high COP velocity during quiet standing and low vestibulo-ocular reflex (VOR) gain (15). If more detailed motion analysis data during quiet standing were available, we might be able to obtain clear characteristics of nGVS effective groups.

In our previous work, we have proposed a method for estimating the center of mass (COM) motion based on the equations of motion of a rigid-body pendulum model from a force platform and inertial measurement unit (IMU) measurements (25). This method can estimate much more information in addition to the COP, body COM displacement, body COM acceleration, head acceleration, head angular velocity, and lower- and upper-body accelerations from practical measurements. In addition, the contribution of the ankle and hip strategies (26) can be derived from the acceleration of the lower and upper bodies. The advantages of this method are as follows: it can evaluate COM displacement and COM acceleration separately as components of COP variation; when COM acceleration is increasing, it can be determined whether the increase is due to stiffness or force variation in COM control; the head acceleration can be evaluated independently; it can be separated whether it is caused by ankle joint strategy or hip joint strategy; and all of the above can be evaluated independently in the anteroposterior (AP) and mediolateral (ML) directions.

In this study, we aimed to evaluate the mechanism of balance changes when nGVS was applied to healthy subjects with their eyes closed during quiet standing on a firm floor. Thirty-two healthy young subjects participated in the experiment, and balance motion during quiet standing was measured using a force platform and an IMU attached to the head. The proposed method can estimate the displacement and acceleration of whole-body COM, head acceleration, and lower- and upper-body COM accelerations, and more detailed motion data can be obtained during quiet standing. This study focuses on the following three issues, which have been unclear in nGVS studies. The first was whether there was a general trend in balance motion according to the five levels of current intensity in the nGVS. We investigated this by comparing the changes in each balance index for all subjects according to current intensity. The second is to clarify the details of the improvement in balance performance in the nGVS effective group. To achieve this, we divided the subjects into three groups based on the indices that suppress the head acceleration and COM sway area with the addition of nGVS, and compared their performance between groups. The third is the prescreening of nGVS effective subjects. We calculated the correlation coefficient between the balance improvement index with nGVS and each balance index without nGVS and investigated the possibility of extracting nGVS effective subjects from the data in quiet standing without nGVS.

## 2 Methods

### 2.1 Estimation method for evaluation variables

**Figure 1:**
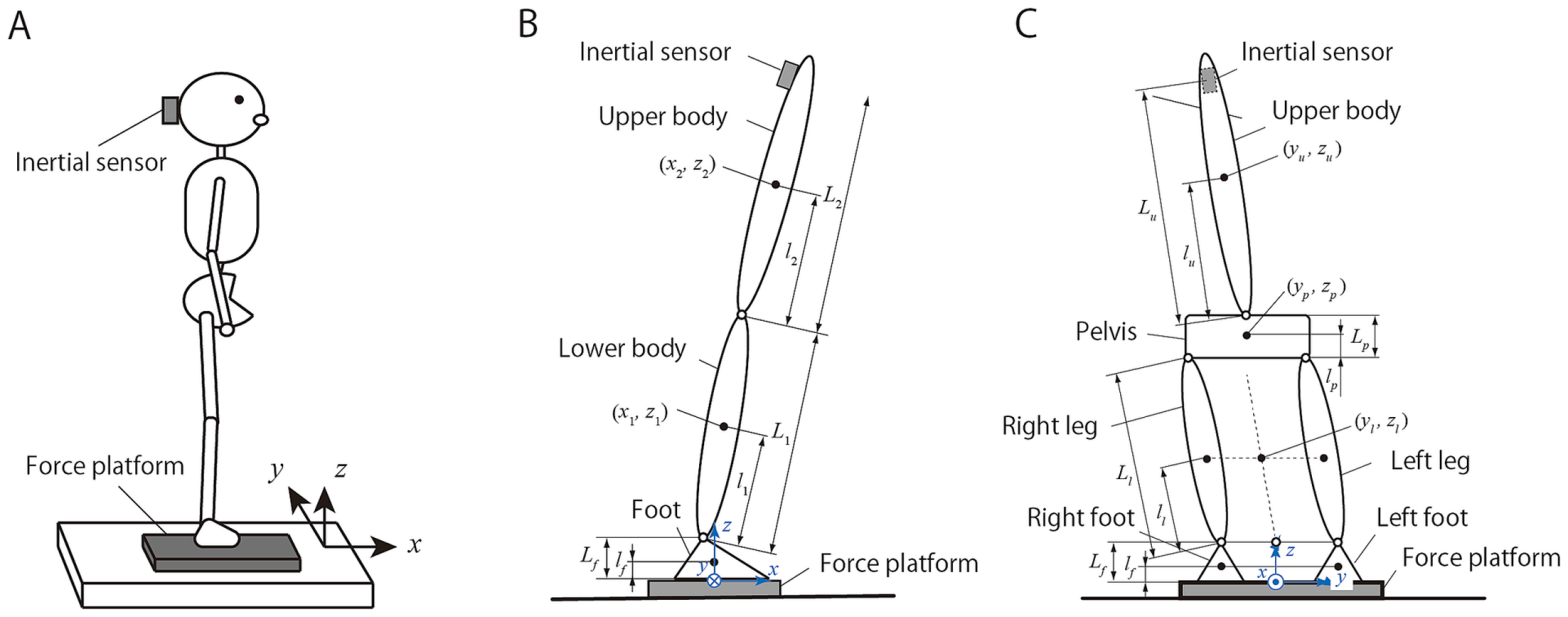
Measurement while standing using a force platform and inertial sensor **(A)**. Two mechanical models were employed to estimate the physical variables for balance evaluation from the measured values: the anteroposterior model composed of the foot, lower body, and upper body **(B)**, and the mediolateral model composed of both feet, both legs, pelvis, and upper body **(C)**.

To measure perturbations during quiet standing, the subject stood on a force platform and an IMU was attached to the back of the head, as shown in Figure 1A. The force platform measures the forces and moments of the force around the triaxes, whereas the IMU measures the acceleration and angular velocity around the triaxes. The coordinate system was determined based on the force platform, with the x-axis in the forward direction, y-axis in the left-hand direction, and the z-axis in the vertically upward direction. The acceleration and angular velocity measured by the IMU were converted from the sensor coordinate system to the coordinate system based on the force platform using attitude angle estimation, excluding the head rotation around the vertical axis because geomagnetism was not used. COM acceleration was estimated by dividing the horizontal force obtained from the force platform by the body weight.

This study estimated the variables for balance evaluation from instrumental values based on the equations of motion of rigid-body link models (25). Different models were defined for the AP and ML directions. In the AP direction, a double pendulum model consisting of feet, lower body, and upper body was assumed, as shown in Figure 1B. The hip joint is defined as the boundary between the lower and upper body. In the ML direction, a multilink model consisting of both feet, both legs, pelvis, and upper body was assumed, as shown in Figure 1C. The legs were assumed to be parallel, making it impossible to consider standing in a wide or narrow stance. Owing to the constraints between the legs and pelvis, the model was regarded as a 2 DOF system consisting of upper and lower bodies whose boundary was near the 5th lumbar vertebra.

Based on the equations of motion of these mechanical models, the COM displacement of the body and accelerations of the lower and upper bodies can be derived in the AP and ML directions using the following equations:


$$\begin{array}{l} \left[\begin{array}{ccc} m_{1} & m_{2} & 0\\ I_{1} & I2 & -m_{b}g\\ \frac{L_{1}}{l_{1}l_{2}}\left(l_{2}-L_{2}\right) & \frac{L_{2}}{l_{2}} & 0 \end{array}\right]\left[\begin{array}{c} {\ddot{x}}_{1}\left(t\right)\\ {\ddot{x}}_{2}\left(t\right)\\ x_{b}\left(t\right) \end{array}\right]=\left[\begin{array}{c} -R_{x}\left(t\right)\\ -N_{y}\left(t\right)\\ {\ddot{x}}_{hd}\left(t\right) \end{array}\right]\\ I_{1}=\frac{J_{1}}{l_{1}}+m_{1}\left(L_{f}+l_{1}\right)-\frac{J_{2}L_{1}}{l_{1}l_{2}},\\ I_{2}=\frac{J_{2}}{l_{2}}+m_{2}\left(L_{f}+L_{1}+l_{2}\right)\text{,} \end{array}$$

$$\begin{array}{l} \left[\begin{array}{ccc} \frac{2m_{l}l_{l}+m_{p}l_{l}}{l_{l}} & m_{u} & 0\\ I_{3} & I_{4} & -m_{b}g\\ \frac{L_{l}}{l_{l}l_{u}}\left(l_{u}-L_{u}\right) & \frac{L_{u}}{l_{u}} & 0 \end{array}\right]\left[\begin{array}{c} {\ddot{y}}_{l}\left(t\right)\\ {\ddot{y}}_{u}\left(t\right)\\ x_{b}\left(t\right) \end{array}\right]=\left[\begin{array}{c} -R_{y}\left(t\right)\\ -N_{x}\left(t\right)\\ {\ddot{y}}_{hd}\left(t\right) \end{array}\right],\\ I_{3}=\frac{2J_{l}l_{u}-J_{u}L_{l}}{l_{u}l_{l}}+2m_{1}\left(L_{f}+l_{l}\right)+\frac{m_{P}L_{l}\left(L_{f}+L_{l}+l_{p}\right)}{l_{l}},\\ I_{4}=\frac{J_{u}}{l_{u}}+m_{u}\left(L_{f}+L_{l}+L_{p}+l_{u}\right)\text{,} \end{array}$$

where *m_i_* is segment mass, *J_i_* is the moment of inertia around the segment COM, *L_i_* is the segment length, and *l_i_* is the COM position from the lower end of the segment. The subscripts denote the segments: *f* is the foot, 1 is the lower body, 2 is the upper body in the AP model, *l* is the leg, *p* is the pelvis, and *u* is the upper body in the ML model. As variables representing standing perturbation, *x_b_* and *y_b_* are the displacements of the body COM, *ẍ*_1_ and *ẍ*_2_ are the AP COM accelerations of the lower and upper body, and *ӱ_l_* and *ӱ_u_* are the ML COM accelerations of both legs and the upper body, respectively. As measured by the force platform, *R_x_* and *R_y_* are the horizontal floor reaction forces on the x and y axes, respectively. *N_x_* and *N_y_* are the moments of force around each axis. The values obtained from the head IMU, i.e., *ẍ_hd_* and *ӱ_hd_*, represent the horizontal head acceleration in each axis.

The process of solving the above equations requires the body parameters. These parameters were approximated from the subject’s height *H* (m) and weight *M* (kg) based on a previous study (27, 28). By substituting these parameters into the above equations, the following equations were obtained according to the subject’s gender.

In the AP direction,


$$\begin{array}{l} \text{Male}\\ \begin{array}{l} \;\left[\begin{array}{ccc} 0.322M & 0.656M & 0\\ 0.0155MH & 0.512MH & -9.59M\\ -2.05 & 2.27 & 0 \end{array}\right]\left[\begin{array}{c} {\ddot{x}}_{1}\left(t\right)\\ {\ddot{x}}_{2}\left(t\right)\\ x_{b}\left(t\right) \end{array}\right]=\left[\begin{array}{c} -R_{x}\left(t\right)\\ -N_{y}\left(t\right)\\ {\ddot{x}}_{hd}\left(t\right) \end{array}\right]\;\\ \text{Female}\;\\ \left[\begin{array}{ccc} 0.352M & 0.626M & 0\\ 0.0253MH & 0.495MH & -9.59M\\ -2.01 & 2.27 & 0 \end{array}\right]\left[\begin{array}{c} {\ddot{x}}_{1}\left(t\right)\\ {\ddot{x}}_{2}\left(t\right)\\ x_{b}\left(t\right) \end{array}\right]=\left[\begin{array}{c} -R_{x}\left(t\right)\\ -N_{y}\left(t\right)\\ {\ddot{x}}_{hd}\left(t\right) \end{array}\right] \end{array}\} \end{array}$$

In the ML direction,


$$\begin{array}{l} \text{Male}\\ \begin{array}{l} \left[\begin{array}{ccc} 0.624M & 0.469M & 0\\ 0.202MH & 0.418MH & -9.59M\\ -2.68 & 2.66 & 0 \end{array}\right]\left[\begin{array}{c} {\ddot{y}}_{l}\left(t\right)\\ {\ddot{y}}_{u}\left(t\right)\\ y_{b}\left(t\right) \end{array}\right]=\left[\begin{array}{c} -R_{y}\left(t\right)\\ N_{x}\left(t\right)\\ {\ddot{y}}_{hd}\left(t\right) \end{array}\right]\;\\ \text{Femlae}\\ \left[\begin{array}{ccc} 0.652M & 0.436M & 0\\ 0.225MH & 0.390MH & -9.59M\\ -2.48 & 2.57 & 0 \end{array}\right]\left[\begin{array}{c} {\ddot{y}}_{l}\left(t\right)\\ {\ddot{y}}_{u}\left(t\right)\\ y_{b}\left(t\right) \end{array}\right]=\left[\begin{array}{c} -R_{y}\left(t\right)\\ N_{x}\left(t\right)\\ {\ddot{y}}_{hd}\left(t\right) \end{array}\right] \end{array}\;\} \end{array}$$

From the above equations, we obtain the COM displacements and accelerations of the lower and upper bodies in the AP and ML directions, respectively. Although a Kalman filter is applied to remove measurement noise in the actual estimation, the details of the procedure are described in the study (25).

### 2.2 Quantification of the impact of joint strategies

This study quantified joint strategies from the COM accelerations of the lower and upper bodies in the AP and ML directions. The joint strategies were composed of an ankle strategy that used ankle joint torque, and a hip strategy that used hip joint torque to control balance. However, the degree of individual joint strategies has not been evaluated, because there are no indices to quantify joint strategies. Our study approximated each strategy based on mechanical models, and evaluated the amplitude and phase relationship of both strategies based on the acceleration of the lower and upper bodies.

We defined the joint strategy as the acceleration ratio (mode) between the lower and upper bodies, neglecting the acceleration caused by gravity. In the ankle strategy mode, the lower and upper bodies moved in a straight line, whereas in the hip strategy mode, the joint torque acted on the hip joint in the AP direction or on the waist joint in the ML direction. To normalize the magnitude of the two joint strategy modes, we used the head acceleration generated by each mode. ${\ddot{\xi }}_{x1},{\ddot{\xi }}_{y1}$ was the head acceleration generated by the ankle strategy, and ${\ddot{\xi }}_{x2},{\ddot{\xi }}_{y2}$ was the head acceleration generated by the hip strategy. The relationships between the COM accelerations of the lower and upper bodies and the modal acceleration of the joint strategies are expressed in the AP and ML directions, as follows:


$$\begin{array}{l} \left[\begin{array}{c} {\ddot{x}}_{1}\\ {\ddot{x}}_{2} \end{array}\right]=\left[\begin{array}{cc} \frac{l_{1}}{L_{1}+L_{2}} & -\frac{\left\{J_{2}+m_{2}l_{2}\left(L_{1}+l_{2}\right)\right\}l_{1}}{D_{1}}\\ \frac{L_{1}+l_{2}}{L_{1}+L_{2}} & \frac{\left(J_{1}+m_{1}l_{1}^{2}\right)l_{2}-J_{2}L_{1}}{D_{1}} \end{array}\right]\left[\begin{array}{c} {\ddot{\xi }}_{x1}\\ {\ddot{\xi }}_{x2} \end{array}\right]\\ D_{1}=\left(J_{1}+m_{1}l_{1}^{2}\right)L_{2}-\left\{J_{2}+m_{2}\left(l_{2}+L_{1}\right)\left(l_{2}-L_{2}\right)\right\}L_{1} \end{array}$$

$$\begin{array}{l} \left[\begin{array}{c} {\ddot{y}}_{l}\\ {\ddot{y}}_{u} \end{array}\right]=\left[\begin{array}{cc} \frac{l_{l}}{L_{l}+L_{u}} & -\frac{\left\{J_{u}+m_{u}l_{u}\left(L_{l}+l_{l}\right)\right\}l_{l}}{D_{2}}\\ \frac{L_{l}+l_{u}}{L_{l}+L_{u}} & \frac{\left(2J_{l}+2m_{l}l_{l}^{2}+m_{p}L_{l}^{2}\right)l_{u}-J_{u}L_{l}}{D_{2}} \end{array}\right]\left[\begin{array}{c} {\ddot{\xi }}_{y1}\\ \xi_{y2} \end{array}\right]\;\\ D_{2}=\left(2J_{l}+2m_{l}l_{l}^{2}+m_{p}L_{l}^{2}\right)L_{u}-\left\{J_{u}+m_{u}\left(l_{l}+L_{l}\right)\left(l_{u}-L_{u}\right)\right\}L_{l} \end{array}$$

After inverting the above equations and substituting the physical parameters, the following equations were obtained according to the gender of the participants:


$$\begin{array}{l} \text{Male}\\ \begin{array}{l} \left[\begin{array}{c} {\ddot{\xi }}_{x1}\left(t\right)\\ {\ddot{\xi }}_{x2}\left(t\right) \end{array}\right]=\left[\begin{array}{cc} 0.00917 & 1.37\\ -2.06 & 0.903 \end{array}\right]\left[\begin{array}{c} {\ddot{x}}_{1}\left(t\right)\\ {\ddot{x}}_{2}\left(t\right) \end{array}\right]\\ \text{Female}\\ \left[\begin{array}{c} {\ddot{\xi }}_{x1}\left(t\right)\\ {\ddot{\xi }}_{x2}\left(t\right) \end{array}\right]=\left[\begin{array}{cc} 0.0343 & 1.36\\ -2.04 & 0.910 \end{array}\right]\left[\begin{array}{c} {\ddot{x}}_{1}\left(t\right)\\ {\ddot{x}}_{2}\left(t\right) \end{array}\right] \end{array}\} \end{array}$$

$$\begin{array}{l} \text{Male}\\ \begin{array}{l} \left[\begin{array}{c} {\ddot{\xi }}_{y1}\left(t\right)\\ {\ddot{\xi }}_{y2}\left(t\right) \end{array}\right]=\left[\begin{array}{cc} 0.512 & 1.06\\ -3.71 & 1.86 \end{array}\right]\left[\begin{array}{c} {\ddot{y}}_{l}\left(t\right)\\ {\ddot{y}}_{u}\left(t\right) \end{array}\right]\\ \text{Female}\\ \left[\begin{array}{c} {\ddot{\xi }}_{y1}\left(t\right)\\ {\ddot{\xi }}_{y2}\left(t\right) \end{array}\right]=\left[\begin{array}{cc} 0.587 & 1.01\\ -3.51 & 1.78 \end{array}\right]\left[\begin{array}{c} {\ddot{y}}_{l}\left(t\right)\\ {\ddot{y}}_{u}\left(t\right) \end{array}\right] \end{array}\} \end{array}$$

The transformation matrices were constant and independent of the subjects’ height and weight. From this definition, the joint strategy mode acceleration satisfies the following equations:


$$\begin{array}{l} {\ddot{\xi }}_{x1}\left(t\right)+{\ddot{\xi }}_{x2}\left(t\right)={\ddot{x}}_{hd}\left(t\right)\\ {\ddot{\xi }}_{y1}\left(t\right)+{\ddot{\xi }}_{y2}\left(t\right)={\ddot{x}}_{hd}\left(t\right) \end{array}\}$$

**Figure 2:**
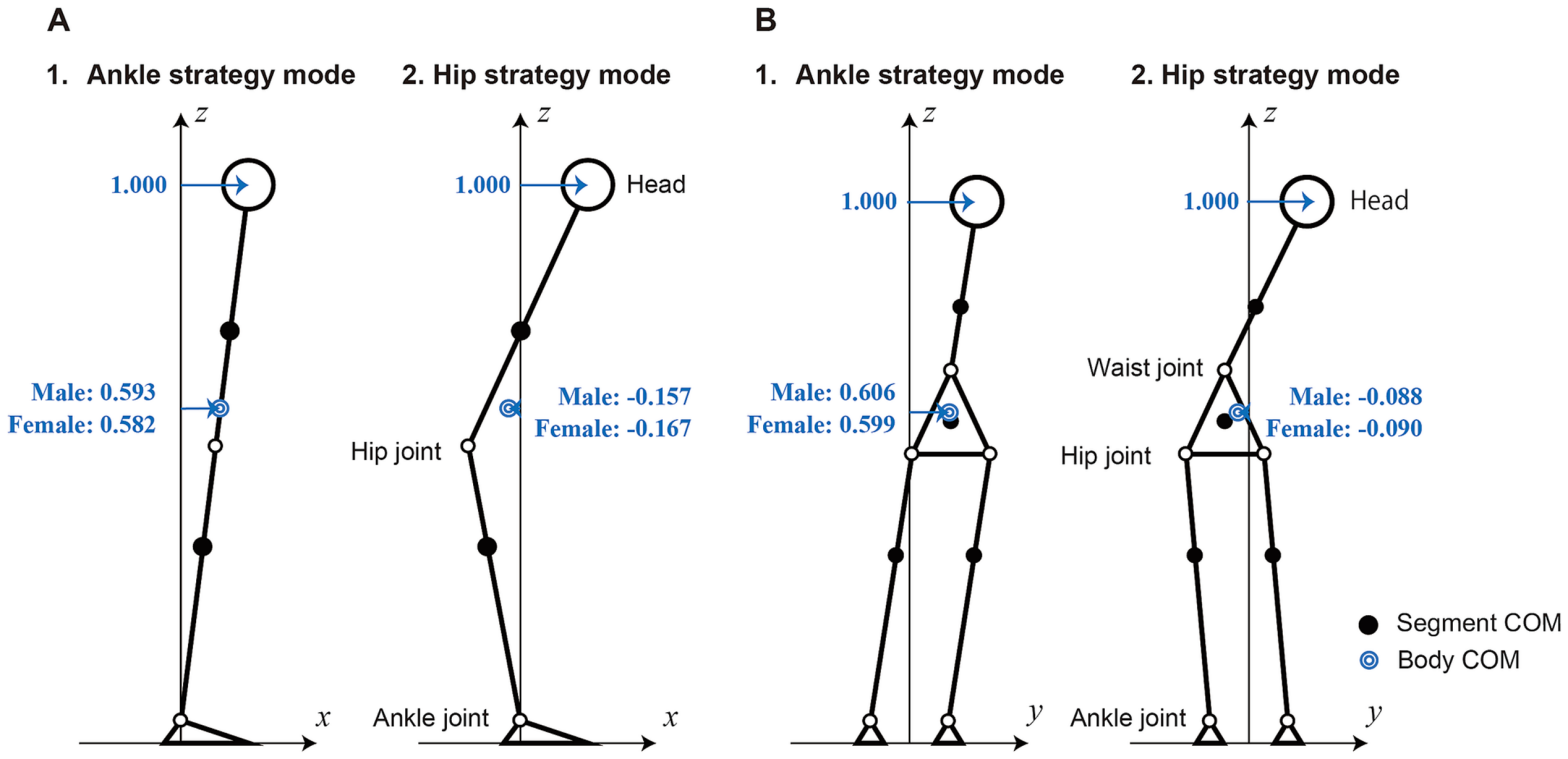
Mode shapes of ankle and hip strategy modes in the AP and ML directions. When the amplitude of the head accelerations is set to one, the ratio of the body COM accelerations is described in the figure. **(A)** Sagittal plane. **(B)** Frontal plane.

These equations indicate that the head acceleration is the sum of the ankle and hip strategy modal accelerations. Figure 2 shows the shapes and magnitudes of the two joint strategy modes. The magnitude of the COM acceleration is shown in blue when the magnitude of the head acceleration was set to one. This result indicates that the contribution of the ankle strategy to COM motion is greater than that of the hip strategy. Consequently, we can discuss COM control using the ankle strategy mode, and head acceleration control using the relationship between the ankle and hip strategy modes.

### 2.3 Balance index

From perturbation measurements during standing using a force platform and head IMU, the COM displacement, COM acceleration, head acceleration, lower body acceleration, and upper body acceleration were estimated in the AP and ML directions. The lower- and upper-body COM accelerations can be transformed into head accelerations arising from the ankle or hip joint strategy. As these variables were direct and useful for evaluating standing balance control, we used them to define the balance index. We removed the offset of the measurements by subtracting the average of the measurements because the offset of the measurements depended on the standing position and characteristics of the instrument.

Table 1 lists the 31 balance evaluation indices used in this study. The subscripts AP and ML indicate indices for the sagittal and frontal planes, respectively. These indices were roughly classified as COP, sway magnitude, COM control, and head acceleration control indices. The conventional COP-based indices were composed of the root mean square (RMS) of the pressure center (RMSCP_AP_, RMSCP_ML_), area of the 95% confidence ellipse of the COP (S95CP), and velocity of the COP trajectory (VELCP).

**Table 1:** Balance evaluation index of 31 indices: classical COP evaluation (four indices), evaluation of body COM and head acceleration sway magnitude (11 indices), evaluation of COM control (eight indices), and evaluation of head acceleration control (eight indices).

Index	Unit	Description
Traditional index based on COP measurement		
RMSCP_AP,_ RMSCP_ML_	mm	Root mean square of COP on each plane
S95CP	mm^2^	95% confidence ellipse area of COP
VELCP	mm/s	COP trajectory velocity
Sway index for COM and head acceleration		
RMSCM_AP_, RMSCM_ML_	mm	Root mean square of COM displacement on each plane
RMSCMA_AP_, RMSCMA_ML_	mm/s^2^	Root mean square of COM acceleration on each plane
S95CM	mm^2^	95% confidence ellipse area of COM
RMSHA_AP_, RMSHA_ML_	mm/s^2^	Root mean square of head acceleration on each plane
RMSHW_AP_, RMSHW_ML_	deg/s	Root mean square of head angular velocity on each plane
AVGHA	mm/s^2^	Mean head acceleration
SR	–	Sway ratio of head acceleration to COM sway area
Index for COM control		
GRAD_AP_, GRAD_ML_	–	The gradient of linear approximation between COM displacement on each plane
IC_AP_, IC_ML_	mm/s^2^	Intercept of linear approximation between COM displacement and acceleration on each plane
SIG_AP_, SIG_ML_	mm/s^2^	The standard deviation of COM acceleration from linear approximation on each plane
CC_AP_, CC_ML_	–	The correlation coefficient between COM displacement and acceleration on each plane
Index for head acceleration control		
RMSXI1_AP_, RMSXI1_ML_	mm/s^2^	Root mean square of head acceleration caused by ankle strategy on each plane
RMSXI2_AP_, RMSXI2_ML_	mm/s^2^	Root mean square of head acceleration caused by hip strategy on each plane
RXI_AP_, RXI_ML_	–	Joint strategy ratio (RMSXI2_AP_/RMSXI1_AP_, RMSXI2_ML_/RMSXI1_ML_)
CXI_AP_, CXI_ML_	–	Correlation coefficient of head acceleration caused by the two joint strategies on each plane

The sway magnitude indices were derived from the motions of the body COM and head considering the control targets. The body COM indices were the RMS of the COM displacement (RMSCM_AP_, RMSCM_ML_), RMS of the COM acceleration (RMSCMA_AP_, RMSCMA_ML_), and area of the 95% confidence ellipse of the COM displacement (S95CM). The head indices were the RMS of head acceleration (RMSHA_AP_, RMSHA_ML_), RMS of head angular velocity (RMSHW_AP_, RMSHW_ML_), mean head acceleration (AVGHA), and sway ratio between the 95% confidence ellipse of the COM displacement and mean head acceleration (SR). The AVGHA was calculated using the following equation:


$$\mathrm{AVGHA}=\frac{1}{N}\overset{N}{\underset{k=1}{\sum }}\sqrt{{\ddot{x}}_{hd}\left(k\right)+{\ddot{y}}_{hd}\left(k\right)}\text{,}$$

where *N* is the total number of sampling data points in the analytical interval, and *k* is the data number. The SR is defined as


$$SR=\mathrm{AVGHA}/\sqrt{S95CM}\text{,}$$

where the denominator determines the square root of the COM area to correct the order of each index.

**Figure 3:**
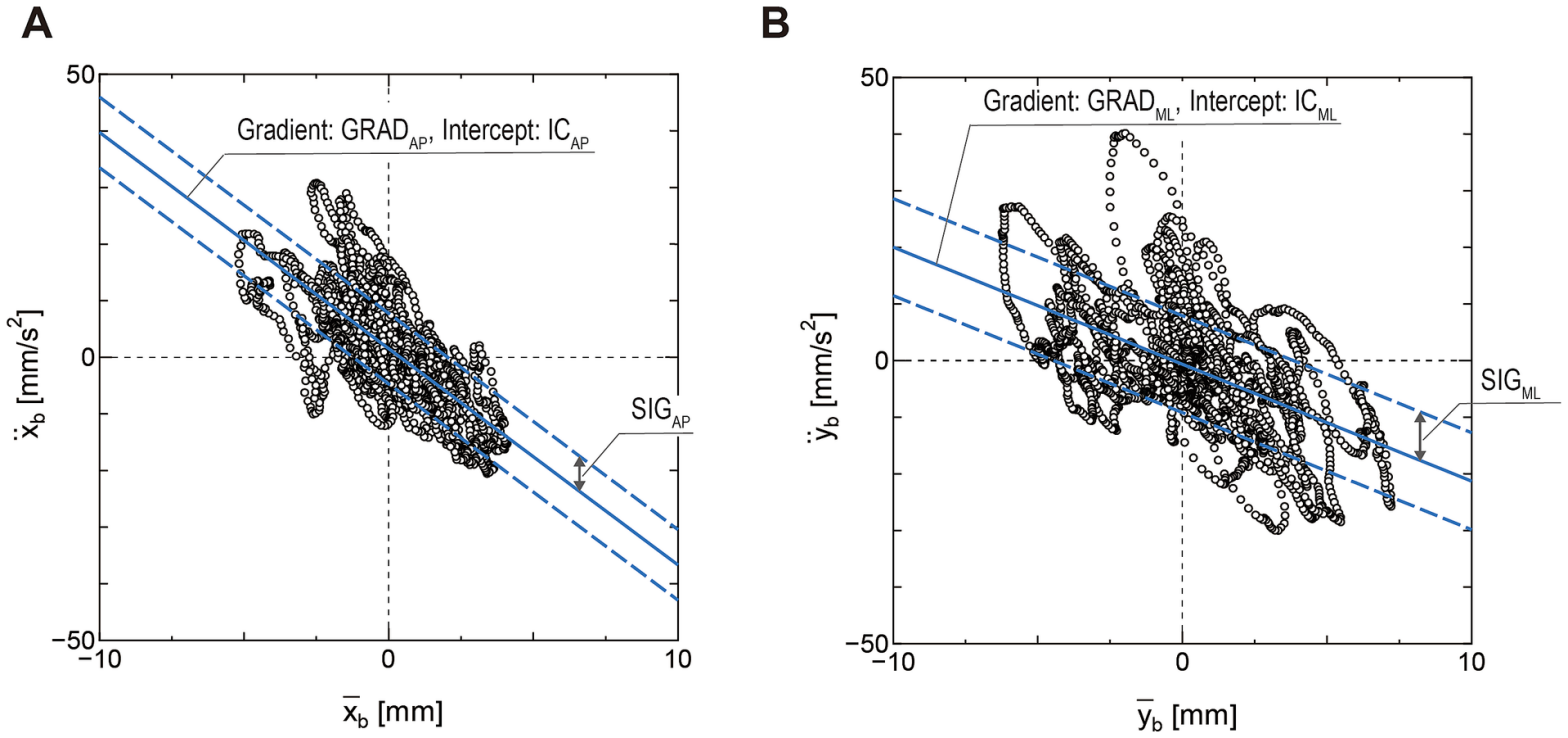
COM displacement (processed with a 0.10 Hz high-pass filter) and COM acceleration plotted in **(A)** AP and **(B)** ML directions as a representative result for the evaluation of COM control. The evaluation indices were the gradient and intercept of the approximate line, and the standard deviation of the COM acceleration from the line.

For COM control indices, we evaluated the relationship between the COM position and recovery force (29). This study evaluated the COM control based on the relationship between the COM displacement and acceleration because the recovery force excited by ankle torque, hip torque, and gravity was proportional to COM acceleration. Notably, the COM displacement contains slow fluctuations at the equilibrium point and fast movements excited by the recovery force (30). To remove the fluctuations of the equilibrium point, we used the COM displacement applied with a high-pass filter with a 0.1 Hz cutoff frequency ( ${\bar{x}}_{b},{\bar{y}}_{b}$ ) in the COM control indices. Figure 3 shows a representative result of the relationship between filtered COM displacement and COM acceleration. We interpreted the gradient of the approximate line (GRAD_AP_, GRAD_ML_) as the stiffness and the intercept (IC_AP_, IC_ML_) as the asymmetry of the recovering force. The standard deviations of the acceleration for the approximated lines (SIG_AP_, SIG_ML_) indicate the variations in the control force. Furthermore, the correlation coefficients between the filtered COM displacement and COM acceleration (CC_AP_, CC_ML_) were considered as indices of the COM control reliability.

The evaluation of the head acceleration control considered the influence of the ankle and hip strategies on head acceleration. We evaluated the RMS of the head acceleration produced by the ankle strategy (RMSXI1_AP_, RMSXI1_ML_) and hip strategy (RMSXI2_AP_, RMSXI2_ML_), and the RMS ratio RMSXI2/RMSXI1 in the AP and ML directions (RXI_AP_, RXI_ML_). When the head acceleration excited by the ankle strategy for COM control may be canceled out by the hip strategy, the correlation coefficients between the modal accelerations of the ankle and hip strategies (CXI_AP_, CXI_ML_) must be negative.

### 2.4 Experiment

The data used in this study were the same as those used in investigations of the optimal intensity of nGVS (13). A total of 32 healthy subjects (16 female, mean age 20.7 ± 0.7 years) with no previous vestibular or neurological disorders participated in the experiment. The study was approved by the Ethical Review Committee of Fukuoka International University of Health and Welfare (Approval No.: 20-fiuhw-011). The heights of the subjects were measured in advance, and their weights were calculated from the floor reaction force measured in the experiment. The subjects were instructed to stand on a force platform with both feet together, their heads facing forward, their arms down naturally, and their eyes closed. There was a 60 s rest period between the measurements under each condition, including the baseline. During the rest period, the subjects maintained a resting standing position with eyes open, without changing the position of the legs.

Force platforms (TF-3040, Tec Gihan, Kyoto, Japan) and a wired inertial sensor (IMS-WD, Tec Gihan) attached to the back of the head were used for measurements. Both measurements were time synchronized with a sampling frequency of 100 Hz. A DC-Stimulator Plus (NeuroCare Group GmbH, Munich, Germany) was used to add the nGVS. The noisy GVS generated a random current level for every sample (sampling rate: 1280 samples/s). Random numbers are normally distributed over time, probability density follows a Gaussian Bell curve, and all coefficients have similar magnitudes in the frequency spectrum (22). The stimulation was composed of five patterns of random noise in the frequency band of 100–640 Hz with maximum noise amplitudes of 50, 100, 200, 300, and 500 μA, in addition to an intensity of 0 μA as a sham stimulus. A 60-s quiet standing test was performed once for each current intensity in random order. The sham stimulus was also given randomly without distinguishing it from the other current intensities. This study analyzed physical sway during the central 40 s of the 60-s period.

### 2.5 Data analysis

As a preprocessing step for the COM estimation, a zero-phase high-pass filter with a cutoff frequency of 0.1 Hz was applied to the horizontal force from the force platform (*R_x_*, *R_y_*) to remove the drift waveform with a low-frequency band. The Kalman filter was used to remove measurement noise to estimate the displacement and acceleration of the body COM, acceleration of the upper and lower body COM, and head acceleration, and the offsets were collected. The 30 s period between 5 and 35 s of the obtained measurements was used as the analysis period. The COP trajectory velocity and sway area have been conventionally used as indices of balance improvement using nGVS; however, it is essential to consider the control of COM displacement and head acceleration. We defined the balance stability transition index (BSTI) by adding nGVS as follows:


$$\mathrm{BSTI}\left(e\right)=\frac{\mathrm{AVGHA}\left(e\right)}{\mathrm{AVGHA}\left(0\right)}\cdot \sqrt{\frac{S95CM\left(e\right)}{S95CM\left(0\right)}}\text{.}$$

The BSTI was described as the ratio of head acceleration to the sham to COM sway area for current intensities (*e*) of 50, 100, 200, 300, and 500 μA for nGVS. Because the COM sway area is a quadratic index, we adjusted it using the square root. If the BSTI score was less than 1, we concluded that the nGVS improved the balance of the subject. The optimal current intensity for each subject was determined as the current, except for 500 μA, at which the BSTI was minimized.

We investigated the overall trend of different intensities of nGVS, the characteristics of the groups divided by effect, and the balance improvement group based on the balance index in sham stimuli. To review the overall trends of different nGVS intensities, the means and standard deviations of all evaluation indices for all subjects at each nGVS intensity were considered. To examine the characteristics of the groups with effects, the subjects were classified into three groups based on the BSTI at the optimal intensity of the nGVS: effective (G1, BSTI < 0.8), moderate (G2, 0.8 ≤ BSTI < 1), and no effect (G3, BSTI ≥ 1.0). The boundary value between effective and moderate was set at 0.8. because the median BSTI for all subjects was 0.8007. Two-way analysis of variance (ANOVA) was applied to groups with and without nGVS, and multiple comparison tests based on the Bonferroni correction were used for indices with significant differences. For this statistical analysis, we used IBM SPSS^®^ (IBM Corporation, Armonk, NY, United States) version 26, with a significance level of *p* < 0.05. To screen the balance improvement group from the balance indices in the sham stimuli, we derived correlation coefficients between the BSTI and indices in the sham stimuli.

## 3 Results

### 3.1 Overall effect of nGVS

**Figure 4:**
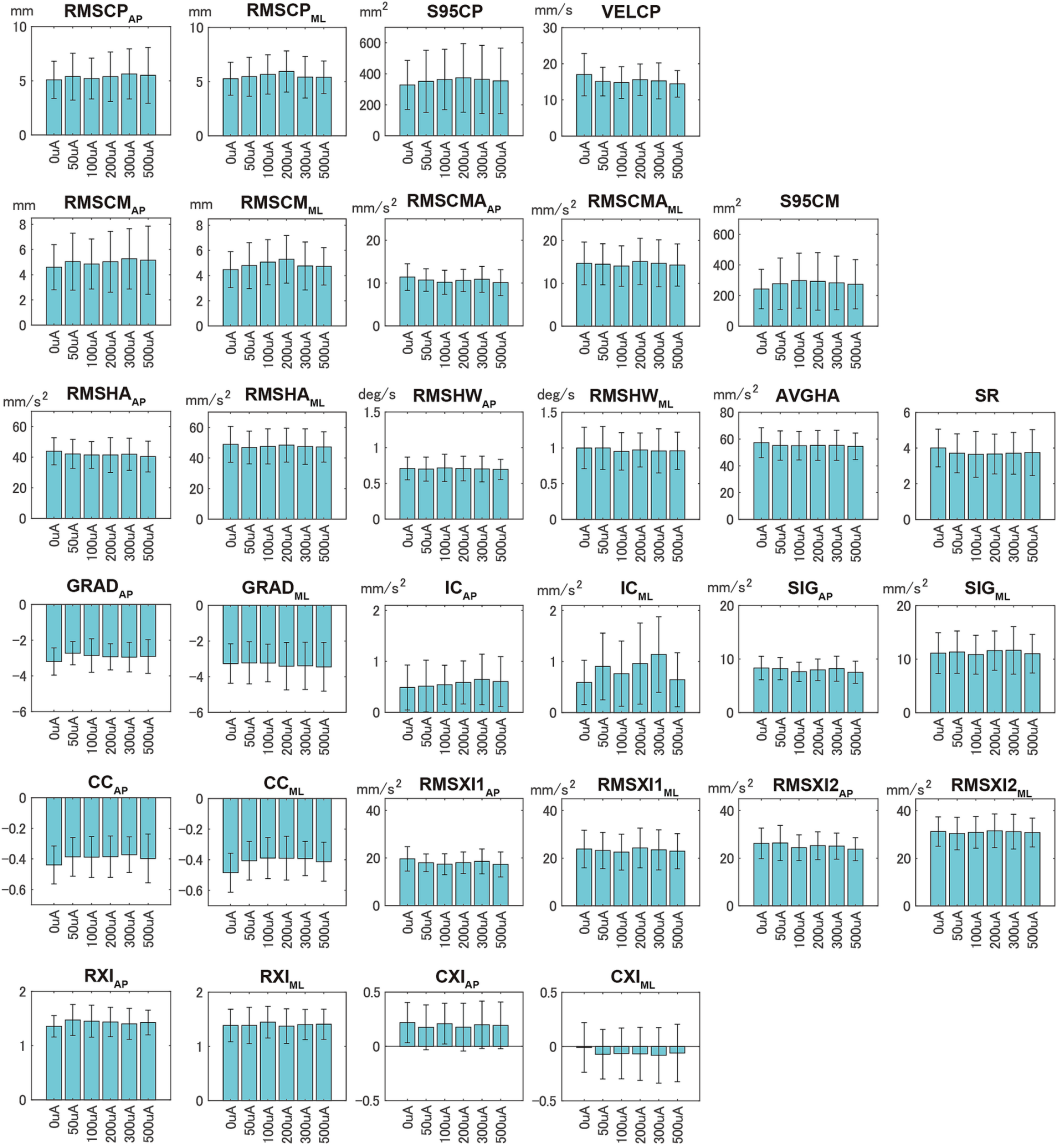
Mean and standard deviation of the balance evaluation indices for all subjects during quiet standing with eyes closed for each current intensity of noisy galvanic vestibular stimulation.

Figure 4 shows the mean and standard deviation of the balance evaluation indices for all subjects for different nGVS intensities (sham and 5 current intensities). No clear characteristics were observed in any of the indices owing to the addition of nGVS. For conventional COP indices, the addition of nGVS tended to increase the 95% confidence ellipse area of the COP (S95CP) and improve the COP trajectory velocity (VELCP). The results indicated that nGVS does not work for all subjects and that the optimal current intensity also differs individually. For the indices introduced in this study, the 95% confidence ellipse area of COM (S95CM) tended to be worse with the addition of nGVS, and peaked at 100–200 uA. This peak trend is similar to the RMS of the COM displacement on the frontal plane (RMSCMML) and the correlation coefficient between COM displacement and acceleration on the frontal plane (CCML), suggesting that the addition of nGVS would increase COM sway on the frontal plane. There was a slight decrease in the average acceleration of the head (AVGHA) with nGVS; however, no clear characteristics were observed according to the current intensities.

### 3.2 Group-based evaluation

Groups G1 and G2 were divided based on the median BSTI of 0.801. There were 16 subjects in G1, nine in G2, and seven in G3. After grouping based on the BSTI for the optimal intensity of the nGVS, we applied a two-way ANOVA to the groups and nGVS stimuli. Table 2 shows the results of the statistical analyses of between-group, between-stimulus, and group-stimulus interactions. Significant differences (*p* < 0.05) are indicated by asterisks for *p* values, and multiple comparison tests were performed. Regarding the differences between the stimuli, significant differences were observed in head acceleration in the AP direction (RMSHA_AP_, *p* = 0.048) and that caused by ankle strategy (RMSXI1_AP_, *p* = 0.045). In contrast, no significant differences were observed in the conventional indices of COP velocity (VELCP, *p* = 0.069) or COP area (S95CP, *p* = 0.343).

**Table 2:** Results of two-way analysis of variance for all balance indices, with and without nGVS stimuli, and for the three groups according to the effects of nGVS.

Index	Group			Stimulation			Group × stimulation		
	*F* value	*P* value	*η_p_* ^2^	*F* value	*P* value	*η_p_* ^2^	*F* value	*P* value	*η_p_* ^2^
RMSCP_AP_	2.094	0.132	0.067	0.702	0.405	0.012	3.713	0.030^*^	0.114
RMSCP_ML_	4.507	0.015^*^	0.135	0.308	0.581	0.005	2.751	0.072	0.087
S95CP	3.199	0.048^*^	0.099	0.914	0.343	0.016	4.741	0.012^*^	0.141
VELCP	3.473	0.038^*^	0.107	3.438	0.069	0.056	2.484	0.092	0.079
RMSCM_AP_	2.355	0.104	0.075	0.214	0.645	0.004	3.318	0.043^*^	0.103
RMSCM_ML_	4.885	0.011^*^	0.144	0.105	0.747	0.002	2.519	0.089	0.080
RMSCMA_AP_	1.549	0.221	0.051	2.211	0.142	0.037	3.574	0.034^*^	0.110
RMSCMA_ML_	3.880	0.026^*^	0.118	0.152	0.698	0.003	2.029	0.141	0.065
S95CM	3.540	0.035^*^	0.109	0.349	0.557	0.006	4.471	0.016^*^	0.134
RMSHA_AP_	0.926	0.402	0.031	4.075	0.048^*^	0.066	0.951	0.392	0.032
RMSHA_ML_	1.811	0.173	0.059	1.252	0.268	0.021	0.069	0.933	0.002
RMSHW_AP_	0.385	0.682	0.013	0.603	0.441	0.010	0.081	0.922	0.003
RMSHW_ML_	1.880	0.162	0.061	1.663	0.202	0.028	0.427	0.654	0.015
AVGHA	1.905	0.158	0.062	3.154	0.081	0.052	0.499	0.610	0.017
SR	2.886	0.064	0.091	0.061	0.805	0.001	3.628	0.033^*^	0.111
GRAD_AP_	1.032	0.363	0.034	3.420	0.069	0.056	0.538	0.587	0.018
GRAD_ML_	0.059	0.942	0.002	0.873	0.354	0.015	0.647	0.528	0.022
IC_AP_	0.568	0.570	0.019	1.464	0.231	0.025	0.299	0.743	0.010
IC_ML_	3.165	0.050	0.098	3.170	0.080	0.052	0.369	0.693	0.013
SIG_AP_	2.295	0.110	0.073	0.546	0.463	0.009	3.357	0.042^*^	0.104
SIG_ML_	5.952	0.004^*^	0.170	0.019	0.890	0.000	2.082	0.134	0.067
CC_AP_	0.768	0.468	0.026	2.698	0.106	0.044	2.398	0.100	0.076
CC_ML_	1.965	0.149	0.063	2.867	0.096	0.047	0.565	0.572	0.019
RMSXI1_AP_	1.482	0.236	0.049	4.203	0.045^*^	0.068	4.191	0.020^*^	0.126
RMSXI1_ML_	2.989	0.058	0.093	0.338	0.563	0.006	1.913	0.157	0.062
RMSXI2_AP_	1.491	0.234	0.049	0.936	0.337	0.016	1.158	0.321	0.038
RMSXI2_ML_	4.359	0.017^*^	0.131	2.115	0.151	0.035	0.481	0.621	0.016
RXI_AP_	0.199	0.820	0.007	2.908	0.094	0.048	1.164	0.319	0.039
RXI_ML_	1.949	0.152	0.063	0.019	0.892	0.000	0.962	0.388	0.032
CXI_AP_	0.323	0.725	0.011	0.984	0.325	0.017	0.208	0.813	0.007
CXI_ML_	4.934	0.010^*^	0.145	2.097	0.153	0.035	0.935	0.399	0.031

An asterisk (*) indicates that a significant difference (*p* < 0.05).

**Figure 5:**
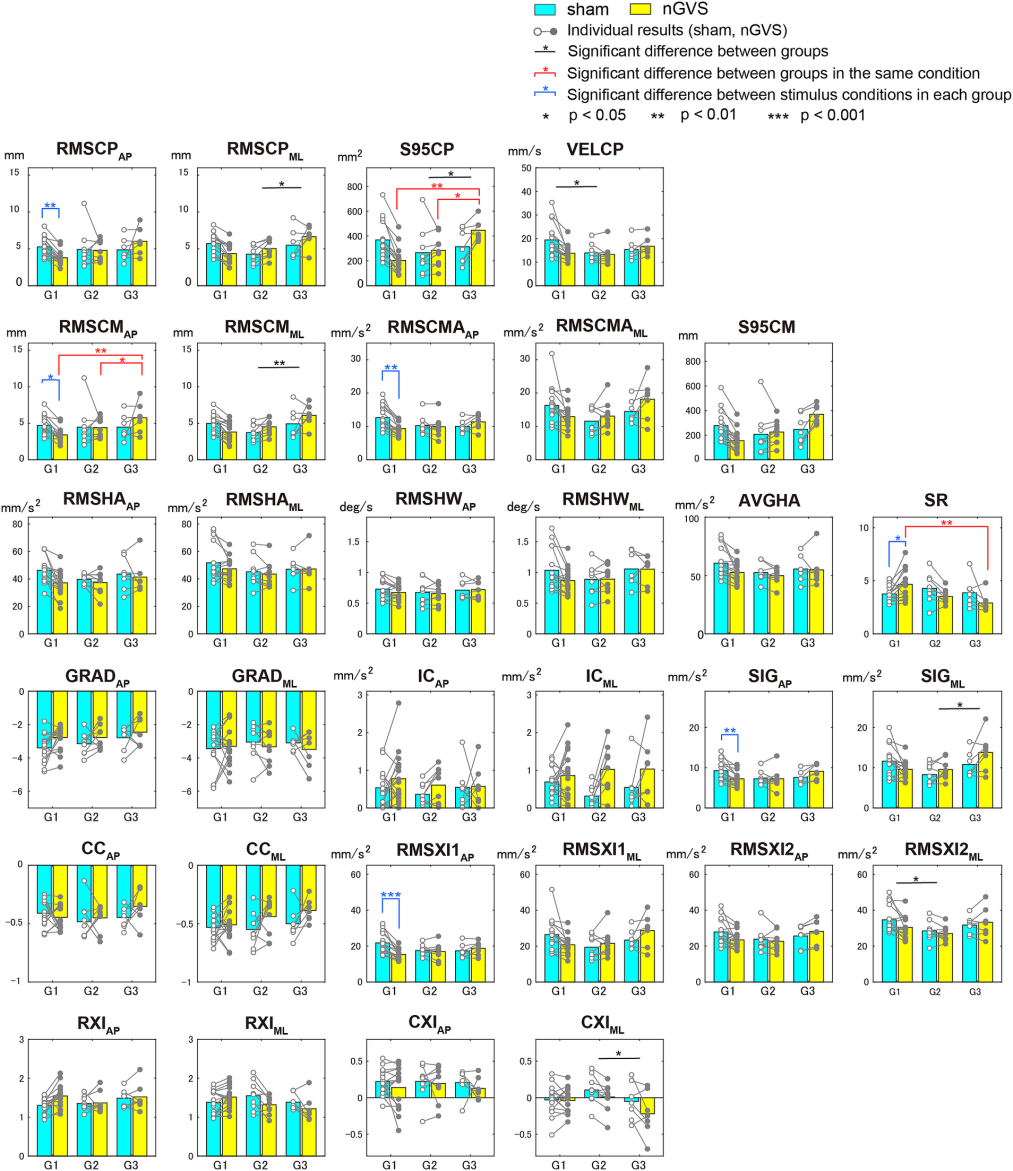
Means and standard deviations of evaluation indices by group according to effects on sham and optimal nGVS stimuli.

Figure 5 shows the means and individual results of each evaluation index for the sham and optimal nGVS groups. Significant differences in multiple comparison tests are indicated with asterisks. Significant differences between the groups are shown in black. There was no evaluation index with a significant difference between G1 and G3. Significant differences between the groups under the same stimulus conditions are shown in red, and found in the COP sway area (S95CP), RMS of COM displacement in the AP direction (RMSCM_AP_), and ratio of head mean acceleration to COM sway area (SR). This result indicates that the classification of groups reflects the magnitude of COP and COM sway rather than head acceleration. The significant differences induced by nGVS within each group are indicated in blue and occurred only in G1, with RMSCP_AP_ (*p* = 0.009), RMSCM_AP_ (*p* = 0.027), RMSCMA_AP_ (*p* = 0.001), SIG_AP_ (*p* = 0.007), and RMSXI1_AP_ (*p* < 0.001) in the AP direction. Because RMSCMA_AP_, SIG_AP_, and RMSXI1_AP_ were related to COM acceleration, the effect of nGVS in G1 could be regarded as a reduction in COM acceleration.

### 3.3 Screening of effective subjects

**Figure 6:**
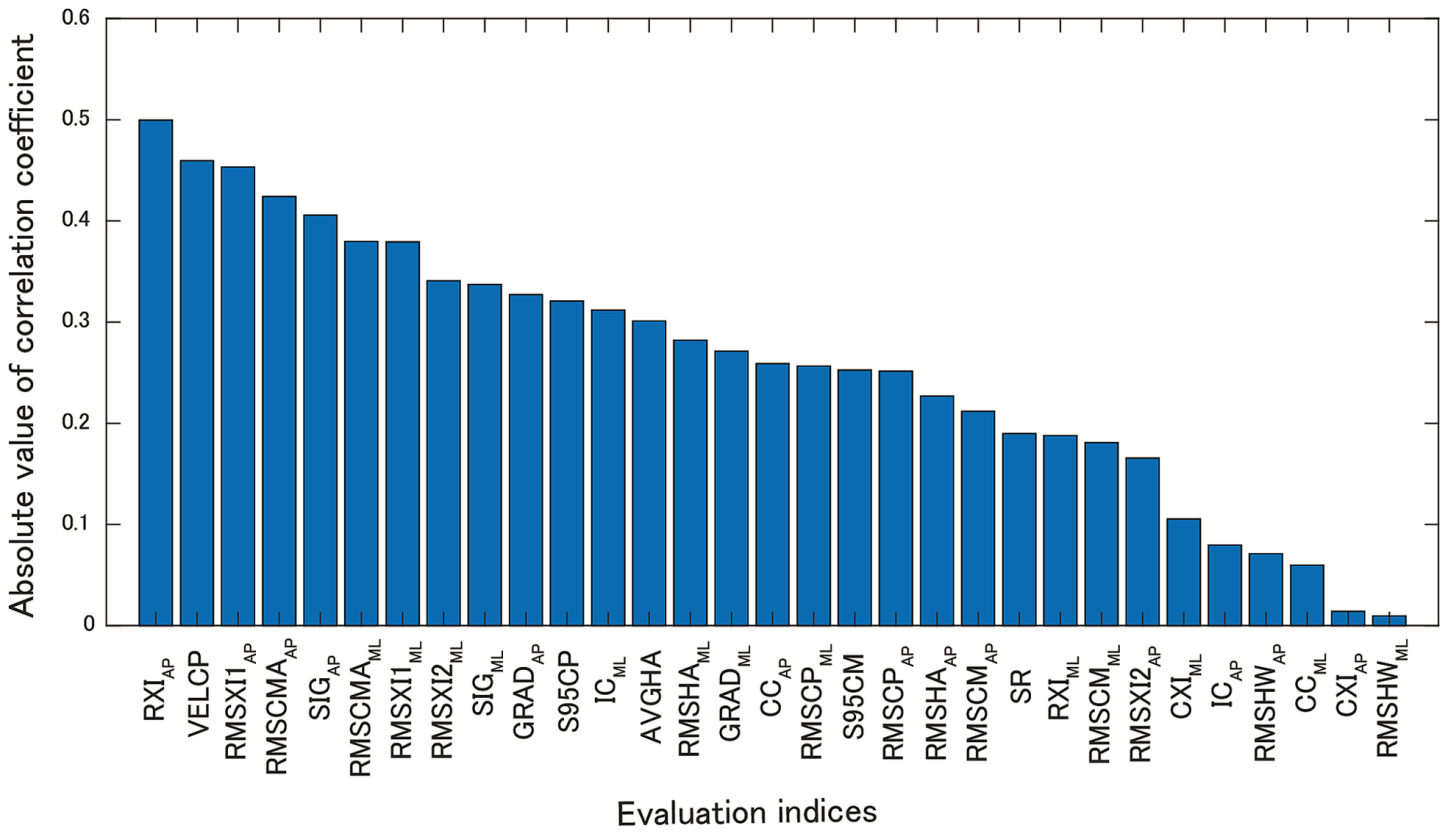
Absolute values of correlation coefficients between BSTI and each index in the sham stimuli.

To screen for effective subjects for nGVS, we calculated the correlation coefficients between the balance evaluation index in the sham stimuli and the BSTI. Figure 6 shows the absolute values of the correlation coefficients (*r*) in the descending order. The results showed that RXI_AP_ (*r* = 0.500), VELCP (*r* = −0.460), RMSXI1AP (*r* = −0.453), RMSCMA_AP_ (*r* = −0.424), and SIG_AP_ (*r* = −0.406) were strongly correlated with BSTI. The effect of nGVS on participants with greater VELCP at baseline was consistent with the results of previous studies (15, 19). The three indices RMSXI1_AP_, RMSCMA_AP_, and SIG_AP_ reflected the acceleration magnitude caused by the ankle strategy, which also affected the VELCP. These results suggest that nGVS is more effective in subjects with higher baseline acceleration variability in the AP direction. The RXI_AP_ had the strongest correlation with BTSI, indicating that the nGVS was more effective when the head acceleration from the ankle strategy was relatively greater than that from the hip strategy.

## 4 Discussion

There were no significant differences in the effects of nGVS for all the subjects, as shown in Figure 4, depending on the current intensity. Although previous studies evaluating the effects of nGVS have investigated the differences in subject groups (subjects with BVP, elderly, or young healthy subjects) and environments (foam or firm floor), the combination of young healthy subjects and firm floors was a difficult condition for detecting significant differences (12, 21). These results indicate that there are individual differences in the effects of nGVS and the optimal current intensity. As a general tendency, the addition of nGVS increased the 95% confidence ellipse area of COM (S95CM) and decreased COP trajectory velocity (VELCP). Because COP is a combined index of COM displacement and COM acceleration (proportional to control force), VELCP decreasing despite increased COM displacement indicates a reduction in COM acceleration. This suggests that the addition of nGVS weakens COM displacement control and improves control force adjustment.

Our study introduced the BSTI, which could evaluate the suppression of COM displacement and head acceleration by the addition of nGVS, and evaluated the subjects by dividing them into three groups. Similar to previous studies, approximately 20% of the participants did not benefit from nGVS. This paper focused on the most effective group (G1) comprised half of the subjects. Their results showed significant differences in the reduction of RMSCP_AP_, RMSCM_AP_, RMSCMA_AP_, SIG_AP_, and RMSXI1_AP_ and an increase in SR between the sham and optimal intensity of nGVS. Most of the above indices were related to COM acceleration, whereas SIG_AP_ and RMSXI1_AP_ were indices for the ankle strategy. Because RMSXI1_AP_ was one of the indices strongly correlated with the COP trajectory velocity (VELCP), the decrease in COP variability in previous studies represented a decrease in ankle strategy-induced head acceleration.

We interpreted that the improvement in vestibular sensitivity reduced SIG_AP_ and RMSXI1_AP_. Vestibular-related reflex function is controlled by sensing from the vestibular organs the head movements in maintaining an upright posture. A previous study (31) showed that nGVS intervention was associated with changes in lateral vestibulospinal tract excitability and body sway. The nGVS intervention could have improved vestibular sensitivity due to increased excitability of vestibular-related nerves, including the vestibulospinal tract. The reason for the difference in the AP direction compared to the ML direction is probably because the base of support is narrow and the balance is unstable. If we can perform the same experiment with a tandem standing (32) or single-leg standing (33), where the ML direction becomes unstable, it is possible that the effect of nGVS can be confirmed in the ML direction as well. This result implies that young healthy subjects use vestibular feedback to control the COM in the AP direction.

Intervention with nGVS requires prescreening of effective subjects because the effects of nGVS vary from individual to individual. A previous study (15) examined the correlation coefficients between the COP trajectory velocity and evaluation indices before the intervention and found high correlations between the COP trajectory velocity (*r* = 0.616) and the VOR gain (*r* = −0.663). This study also calculated the correlation coefficients between the BSTI and evaluation indices in sham stimuli and found that RXIAP (= RMSXI2_AP_/RMSXI1_AP_) had the highest correlation (*r* = 0.500), whereas VELCP had the second highest correlation (*r* = −0.460). Although the absolute values of the correlation coefficients were lower than those reported in a previous study (15), the reason was that the participants in this study were healthy young subjects. In addition, the correlation coefficients for RMSXI1_AP_, RMSCMA_AP_, and SIG_AP_ were large. Therefore, our results support the findings of Wuehr et al. (15), and suggest that nGVS is effective for subjects with large acceleration of COM and head in the AP direction, which is a reason for the COP trajectory velocity being effective for pre-screening.

This study had some limitations. For COM control, we mainly evaluated the stiffness and control accuracy; however, the damping and time delay were neglected. Although time delay has a significant effect on balance stability and is generally determined to be 100–200 ms (34), its identification from quiet standing data is difficult. We also evaluated the effect of the nGVS intervention on balance improvement with the BSTI, but included a COM sway area with poor reproducibility; thus, there is room for further improvement in the evaluation of COM sway. There is no knowledge of the carryover effect in nGVS. Although this experiment was implemented on the same day, this could have affected the results. For the statistical analysis, we have not verified the similarity and importance of the parameters by dimensionality reduction analysis including principal component analysis. While this study improves our understanding of the balance changes mechanism by nGVS with clustering of each parameter, further statistical analysis should be considered in future work, such as dimensionality reduction analysis. For the measurement, it is difficult to estimate the COM movement during standing on the foam floor, because the subject’s sway is estimated via a mechanical model. Previous studies have shown that the effect of nGVS was clear when standing on foam (12, 35). However, we could not evaluate the balance ability in this environment because we estimated COM displacement using the equations of motion of the mechanical model.

The results of our study were limited to healthy young participants. The improvement in balance with nGVS was apparent in the AP direction; however, significant differences might also occur in the ML direction in subjects with BVP who have a large sway in the ML direction (36). The advantage of our evaluation method is that it is easy to measure and the evaluation value can be obtained immediately after measurement, so it can be used to investigate the optimal current intensity of nGVS for individuals and to screen the effective subjects for nGVS.

## 5 Conclusion

In this study, we evaluated the effect of nGVS based on control performance during quiet standing with eyes closed on COM displacement and head acceleration using a force platform and inertial sensor. The results showed that the present method is useful for understanding how the nGVS effective group improved their balance during quiet standing. The following three findings were obtained: First, the addition of nGVS did not provide a common benefit for all subjects. Second, subjects who improved their balance with nGVS showed a dominant improvement in force control in the AP direction using the ankle joint strategy. Furthermore, we found that the nGVS-effective subjects had a large COM acceleration in the AP direction in the initial quiet standing with eyes closed.

## Data Availability

The data analyzed in this study is subject to the following licenses/restrictions: there are no particular restrictions. Requests to access these datasets should be directed to Motomichi Sonobe, sonobe.motomichi@kochi-tech.ac.jp.
